# Measurement of Warfarin in the Oral Fluid of Patients Undergoing Anticoagulant Oral Therapy

**DOI:** 10.1371/journal.pone.0028182

**Published:** 2011-12-02

**Authors:** Silvia Ghimenti, Tommaso Lomonaco, Massimo Onor, Laura Murgia, Aldo Paolicchi, Roger Fuoco, Lucia Ruocco, Giovanni Pellegrini, Maria Giovanna Trivella, Fabio Di Francesco

**Affiliations:** 1 Dipartimento di Chimica e Chimica Industriale – Università di Pisa, Pisa, Italy; 2 Istituto di Chimica dei Composti Organometallici – CNR, Pisa, Italy; 3 Dipartimento di Patologia Sperimentale BMIE, sez. Patologia Generale e Clinica – Università di Pisa, Pisa, Italy; 4 Laboratorio di Analisi Chimico – Cliniche - Azienda Ospedaliero Universitaria Pisana, Pisa, Italy; 5 Istituto Fisiologia Clinica – CNR, Pisa, Italy; Leiden University Medical Center, Netherlands

## Abstract

**Background:**

Patients on warfarin therapy undergo invasive and expensive checks for the coagulability of their blood. No information on coagulation levels is currently available between two controls.

**Methodology:**

A method was developed to determine warfarin in oral fluid by HPLC and fluorimetric detection. The chromatographic separation was performed at room temperature on a C-18 reversed-phase column, 65% PBS and 35% methanol mobile phase, flow rate 0.7 mL/min, injection volume 25 µL, excitation wavelength 310 nm, emission wavelength 400 nm.

**Findings:**

The method was free from interference and matrix effect, linear in the range 0.2–100 ng/mL, with a detection limit of 0.2 ng/mL. Its coefficient of variation was <3% for intra-day measurements and <5% for inter-day measurements. The average concentration of warfarin in the oral fluid of 50 patients was 2.5±1.6 ng/mL (range 0.8–7.6 ng/mL). Dosage was not correlated to INR (r = −0.03, p = 0.85) but positively correlated to warfarin concentration in the oral fluid (r = 0.39, p = 0.006). The correlation between warfarin concentration and pH in the oral fluid (r = 0.37, p = 0.009) confirmed the importance of pH in regulating the drug transfer from blood. A correlation between warfarin concentration in the oral fluid and INR was only found in samples with pH values ≥7.2 (r = 0.84, p = 0.004).

**Conclusions:**

Warfarin diffuses from blood to oral fluid. The method allows to measure its concentration in this matrix and to analyze correlations with INR and other parameters.

## Introduction

Oral anticoagulants are used when an accurate control of coagulation is required, for example in conditions such as pulmonary embolism and atrial fibrillation. Warfarin, the most common anticoagulant [1], has a narrow therapeutic index [2] and is prone to interference from diet and other drugs [3]–[6]. For these reasons, blood coagulability requires frequent monitoring, typically every day when treatment is started, then once every two weeks or once a month when a stable dose-response relationship has been obtained [7]-[8].

A main problem arising from the adoption of the International Normalized Ratio (INR) as the standard coagulation assay [9] is the social and economic cost relating to the frequent access of patients to laboratories and anticoagulation clinics. The introduction of instruments that enable patients to monitor INR values from a blood drop at home represents a first step towards a solution. Studies suggest that home monitoring with the remote supervision by a physician or an anticoagulation service can improve the accuracy of anticoagulation control and prevent adverse events [10]–[14].

Therapeutic drug monitoring in oral fluid is not a new concept [15]–[17] however no study has been published to our knowledge concerning warfarin or other anticoagulants. Several mechanisms have been identified for the transfer of analytes from blood to oral fluid, whose efficiency is reflected by the oral fluid to plasma concentration ratio (OF/P ratio) [18]-[19]. Factors such as molecular weight, protein binding (∼99% of warfarin in plasma is bound to albumin [20]), elimination kinetics, solubility in water and/or lipids may influence this ratio. In the case of ionizable drugs, the inclination of the ionic and the neutral form to pass from the blood to oral fluid may be drastically different. For this reason, the pH values of these fluids are key factors for determining equilibria and transfer rates. A theoretical OF/P (oral fluid to plasma) concentration ratio can be estimated from the Rasmussen equation [21].

The concentration of a drug in oral fluid essentially represents the free nonionized fraction of the drug in plasma, which is the pharmacologically active fraction. Thus, an analysis of oral fluid has the potential for minimally invasive monitoring of the active fraction of drugs in a far less complex chemical matrix than blood. In this work a method to determine the concentration of warfarin in oral fluid is presented. This method was also successfully applied to a few sweat samples (Fig. S1). The concentration of the drug and pH were measured in the oral fluid samples collected from 50 patients and their correlations to INR and dose were evaluated.

## Materials and Methods

### Ethics statement

Ethical permission was obtained from the Ethics Committee of the Azienda Ospedaliero-Universitaria Pisana. All patients and nominally healthy subjects who volunteered to join the project gave written informed consent prior to their participation.

### Study subjects

Fifty adults (27 males, 23 females) on warfarin therapy were recruited. Patients with hepatic or kidney pathologies were excluded from the study. The enrolled subjects were treated for atrial fibrillation (AF, 62%), deep vein thrombosis (DVT, 12%), pulmonary embolism (PE, 10%), as mechanical or biological heart valve bearers (MHV, 10%) or for other reasons (6%). Their mean age was 74±9 years (range, 42–88 years) and they were on an average warfarin dose of 27±13 mg/week (range, 5–57.5 mg/week). INR values varied from 1.2 to 3.8, with an average value of 2.3±0.6. Approximately three fifths of the patients (31) had INR values within the recommended range for anticoagulation (2.0–3.0). The Mann-Witney test did not highlight statistically significant gender differences (p<0.05) for any of the above parameters. Ten nominally healthy subjects who were not taking any drugs also contributed to the project by providing control samples.

### Chemical reagents

Warfarin, i.e. 3-(α-acetonylbenzyl)-4-hydroxycoumarin sodium salt (purity ≥98%), sodium phosphate monobasic (purity ≥99.0%), potassium phosphate dibasic (purity ≥99.0%) and high-performance liquid chromatography (HPLC) grade methanol were purchased from Sigma Aldrich. HPLC grade water was produced by a Milli-Q Reagent Water System (Millipore, USA). The thromboplastin reagent (HemosIL RecombiPlasTin 2G), thromboplastin diluent (HemosIL RecombiPlasTin 2G Diluent), calibration plasma, normal control assay, low and high abnormal control assays used for INR measurements and quality control were supplied from the Instrumentation Laboratory (Milan, Italy).

A stock solution (1 M, pH 7.02) was prepared by dissolving sodium phosphate monobasic and potassium phosphate dibasic in water. This solution was then diluted to 25 mM to obtain the phosphate buffer solution (PBS) used for HPLC analyses.

A 1 mg/mL stock solution of warfarin was prepared by dissolving the weighed pure compound in water and then diluted with PBS to prepare a 10 µg/mL standard solution. This solution was further diluted with PBS to achieve 0.5, 1, 2, 5, 10, 20, 50 and 100 ng/mL standard working solutions of warfarin.

### Instrumentation

High performance liquid chromatography was carried out by a Jasco HPLC system equipped with an autosampler (AS 2055), a quaternary low pressure gradient pump (PU 2089) and a spectrofluorimetric detector (FP 2020). The column temperature was controlled by a thermostat (HT 3000, ClinLab). The HPLC system was controlled by ChromNAV™ software from Jasco. To validate the analytical method, a few selected samples were also analyzed by a Micro HPLC system (Perkin Elmer Series 200) coupled with an autosampler (Series 200) and a triple quadrupole mass spectrometer (Applied Biosystems Sciex API 365) operating in the Selected Reaction Monitoring (SRM) mode. The mass spectrometer was equipped with an Atmospheric Pressure Photo-Ionization (APPI) interface operating in negative ion mode. The INR measurements were carried out by an automatic system (ACL TOP700, Instrumentation Laboratory) equipped with an autosampler. Data processing was performed by Prism 5 (GraphPad Software Inc.).

### Experimental conditions

Chromatographic separation by the Jasco HPLC system was carried out in isocratic conditions with two different columns:

a C-18 reversed-phase column Chromspher 5 PAH, Varian, 150×4.6 mm, 5 µm, coupled with a guard column ChromSep SS 10×2 mm, Varian, a 72% PBS and 28% methanol mobile phase at a flow rate of 1.0 mL/min; injection volume 25 µL, temperature 25°C;
a C-18 reversed-phase column Poroshell 120 EC-C18, Agilent Technologies, 100×4.6 mm, 2.7 µm connected to a guard column TC - C18, 12.5×4.6 mm, 5 µm, Agilent Technologies, with a 65% PBS and 35% methanol mobile phase at a flow rate of 0.7 mL/min, injection volume 25 µL, temperature 25°C.

Spectrofluorimetric measurements were performed at an excitation wavelength of 310 nm and an emission wavelength of 400 nm. The total run time was 15 minutes.

Chromatographic separation by the Perkin Elmer HPLC system was carried out in isocratic conditions on a C-18 reversed-phase column Chromspher 5 PAH, Varian, 150×4.6 mm, 5 µm, coupled with a guard column ChromSep SS 10×2 mm, Varian. A 50% acetonitrile, 50% water containing 0.1% formic acid mobile phase was used at a flow rate of 1 mL/min at 25°C. The injection volume was 25 µL. Warfarin was quantified by the mass spectrometric detector observing the following two ion transitions (expressed by their mass number/charge number ratios): m/z 307→161 and m/z 307→250.

Spectrometric INR measurements were carried out at a wavelength of 671 nm to minimize optical interference (e.g. hemoglobin and bilirubin). The quality control procedure for INR measurements consisted in analysing three reference samples (normal, low and high INR levels) provided by the Instrumentation Laboratory at least once every eight hours. A coefficient of variation <1% for measurements performed on the same day and <3% for measurements performed on different days demonstrated the good repeatability of the method.

### Sample collection and preparation

Roll-shaped biocompatible synthetic swabs (Salivette® Cortisol, Sarstedt) were delivered to the study subjects to be kept in the mouth for 5 min. Oral fluid was then recovered by centrifugation of the swabs at 3000 rpm for 5 min, filtered by 0.2 µm syringe filters (Spartan, Whatman) and stored at 4°C until assay. Before storage, the pH value was measured by narrow range (resolution 0.3 pH units) pH paper strips (Pehanon, Macherey Nagel).

All samples were collected at the same time (8.30–10.00 AM) to avoid any effects due to possible circadian variations. Patients generally take warfarin in the late afternoon, therefore it can be assumed that the last dose had been completely absorbed when samples were collected.

Pooled patient oral fluid samples (POFSs) were obtained by pooling samples from 20 patients. Control oral fluid samples (COFSs) were obtained from 10 volunteers who stated that they were not taking warfarin. Aliquots of COFS were spiked with known amounts of warfarin to obtain standard oral fluid samples (SOFSs) at different concentration levels.

## Results and Discussion

### Method validation

**Figure 1:**
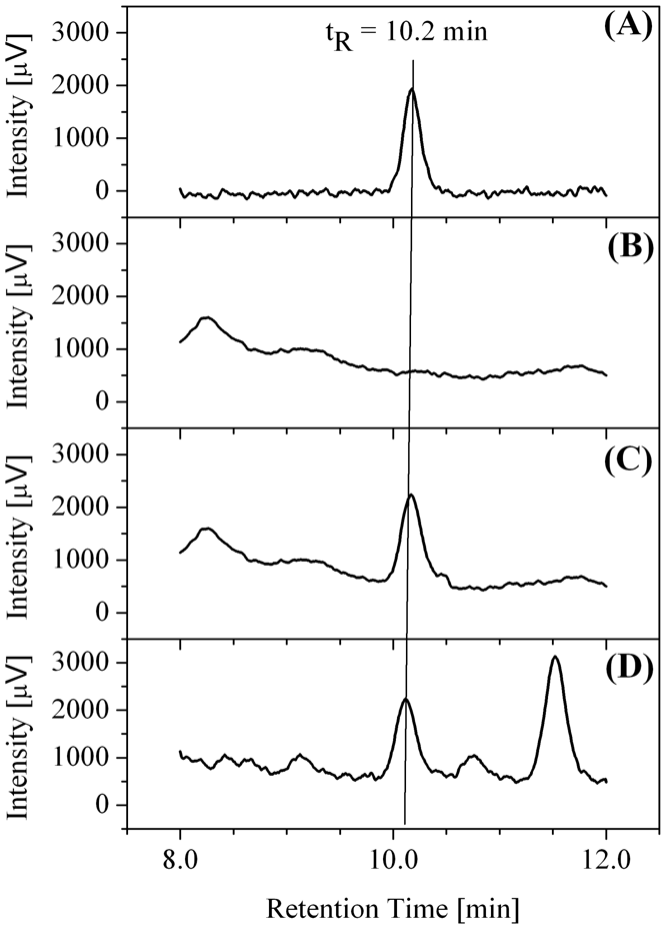
HPLC chromatograms of: A) a standard working solution with a warfarin concentration of 1.5 ng/mL; B) a control oral fluid sample (COFS); C) a standard oral fluid sample (SOFS) with a warfarin concentration of 1.4 ng/mL; D) a patient oral fluid sample (POFS) with a warfarin concentration of 1.4 ng/mL. (Chromatographic conditions: Poroshell 120 EC-C18 column, 65% PBS and 35% methanol mobile phase, flow rate of 0.7 mL/min; injection volume 25 µL).

Figure 1 shows the HPLC chromatograms of a warfarin standard solution, a COFS, a SOFS and a POFS. The standard solution, the SOFS and the POFS had approximately the same warfarin concentrations. The warfarin peaks had a symmetric shape and were well separated from the other chromatographic signals. In the experimental conditions reported in the caption, the retention time (t_R_) was 10.2 min with a standard deviation of 0.1 min in five replicate measurements. PBS blanks, standard working solutions and SOFSs were randomly included within the sequences of analyses of patients' samples for quality control. The method validation included the evaluation of interferences, matrix effect, calibration curve, limit of detection (LOD), intra-day and inter-day precision, stability and recovery.

### Interference

The possible presence of interfering endogenous substances was investigated by comparing the warfarin concentration determined in a standard solution containing 5.0 ng/mL, a SOFS with a spiked concentration of 5.0 ng/mL and two POFSs with unknown concentrations. The samples were analyzed in triplicate, by HPLC-Fluorimetry with both columns (i.e. Chromspher 5 PAH and Poroshell 120 EC-C18) and by HPLC-MS/MS. As already stated, the latter technique was only used to confirm and validate the results of the former.

Table 1 shows the warfarin mean concentration and the coefficient of variation for all the samples. These results highlighted a very good agreement between the various methods in the case of the standard solution and the SOFS, both with an expected value of 5.0 ng/mL. The difference between the concentrations measured with HPLC-MS/MS and HPLC-Fluorimetry was about 10%. The situation was quite different for the two samples collected from patients under oral anticoagulant therapy. The concentration values obtained by the HPLC-MS/MS, which were assumed as reference values, and by the HPLC-Fluorimetry with Poroshell 120 EC-C18 were in good agreement (+9% for POFS-1 and +12% for POFS-2), whereas the values obtained by the HPLC-Fluorimetry with Chromspher 5 PAH column were much higher (+40% for POFS-1 and +76% for POFS-2). The interference, which was only present in the patients' samples, may result from the presence of a warfarin metabolite [22]. On the basis of these results, the Poroshell 120 EC-C18 column was used in the subsequent evaluations of the performance of the method and in the clinical study.

**Table 1:** Comparative determination of warfarin in a standard solution, SOFS and POFS by HPLC-Fluorimetry with both columns (Chromspher 5 PAH and Poroshell 120 EC-C18) and by HPLC-MS/MS.

Sample	Warfarin mean concentration (n = 3), ng/mL (CV%)			
	Expected value	HPLC-Fluo (Poroshell)	HPLC-Fluo (Chromspher)	HPLC-MS/MS
Standard solution	5.0	4.6 (5%)	4.9 (3%)	5.2 (3%)
SOFS	5.0	5.3 (6%)	5.0 (4%)	4.9 (4%)
POFS-1	unknown	3.5 (6%)	4.5 (5%)	3.2 (7%)
POFS-2	unknown	7.1 (4%)	11.1 (4%)	6.3 (6%)

### Matrix effect

The evidence that the shape and position of the warfarin peaks were not affected by the presence of the oral fluid matrix (Fig. 1) suggested the absence of any notable matrix effect. This hypothesis was confirmed by the limited difference of the slopes of the calibration curves obtained in the 1–10 ng/mL concentration range for a set of standard solutions and a set of spiked POFSs, which were 19380±60 and 19400±300, respectively. The slopes values were compared by testing the null hypothesis and their difference resulted not significant (P = 0.87, two-tailed).

### Calibration curve

The warfarin peak area linearly increased with the warfarin concentration of standard working solutions in the observed concentration range 0.5–100 ng/mL. According to Deming regression analysis, the best fit model was: y = (19380±60) x, (R^2^ = 0.9999, standard deviation of residuals sy.x = 1000 AU).

### Limit of detection

A SOFS at about 0.5 ng/mL was prepared and assumed as “blank” or the closest concentration to the limit of detection. This sample was analyzed five times and the corresponding standard deviation (sb) was 0.07 ng/mL. According to the IUPAC definition [23], the LOD was evaluated as three times the standard deviation sb of the “blank”, which corresponds to about 0.2 ng/mL.

### Precision

Within-run repeatability was estimated in SOFSs with a warfarin concentration of 1.0, 5.2 and 10.1 ng/mL. The correspondent relative standard deviations obtained in 6 replicate measurements were 6, 4 and 2% respectively. A total standard deviation of 8% was estimated in a SOFS with a warfarin concentration of 2.9 ng/ml which was analyzed 30 times in six weeks.

### Recovery and stability

The recovery percentage of warfarin from the sampling swabs was estimated at three pH levels (6, 6.9, 7.5) covering the range of variability observed in the oral fluid samples. Three SOFSs with a warfarin concentration of about 5 ng/mL, a volume of 4 mL and an initial pH value of 6.9 were used for the experiments. One was acidified to pH 6 by adding 10 µL of phosphoric acid 1 M, another was alkalinized to pH 7.5 by adding 8 µL of sodium hydroxide 1 M. Each of these three samples was divided into four aliquots. The first aliquot was analyzed without any further treatment, the other three were absorbed into the synthetic swabs and treated as normal samples. The recovery percentage was then calculated from the ratio between the average warfarin concentration in the samples recovered from the swab and the initial concentration. The recovery percentage of the filtration step was estimated in the same way.

The recovery of warfarin from the sampling swabs was satisfactory. No dependence on pH was found, with recovery percentages equal to 99±0.1% at pH 6 and 100±0.1% at pH 6.9 and 7.5. Experiments performed to assess warfarin recovery during the filtration step showed a value of 99±2% at a concentration level of 5 ng/mL.

The stability in time of the treated samples (i.e. centrifuged and filtrated) was assessed in aliquots of POFS during a one and half month storage at 4°C. No significant degradation occurred.

### Clinical Study

The average concentration of warfarin in the oral fluid was 2.5±1.6 ng/mL, with values ranging from 0.8 to 7.6 ng/mL, whereas the pH of the oral fluid was 6.7±0.4 in the range 6.0–7.5 (Fig. S2).

**Figure 2:**
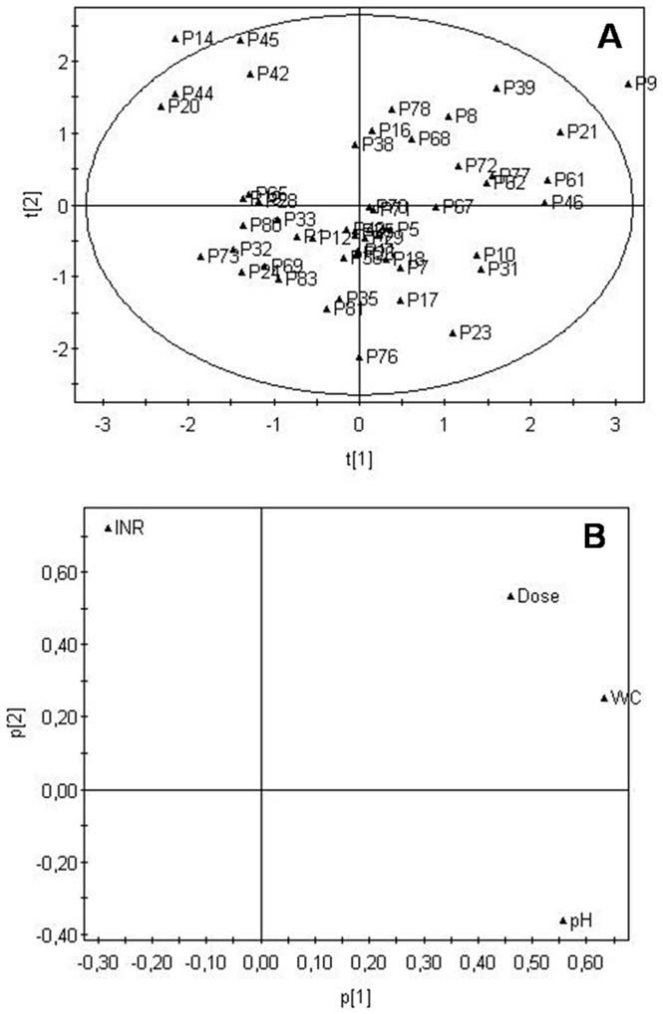
Principal component analysis of patient data. A) score plot, B) loadings plot. Legend: pH of the oral fluid (pH), warfarin concentration in the oral fluid (WC), warfarin dose (Dose), INR.

Principal component analysis [24]-[25] was used to obtain an overall view of the internal structure of the data (Fig. 2). The principal component analysis produces two plots in which similar items are located close to one another: the score plot (Fig. 2A) shows the relationships between the objects, and the loadings plot (Fig. 2B) shows the correlation between the variables. If the two plots are superimposed, the objects characterized by a high value of a variable will be located close to this variable. In our case, the score plot shows that the majority of patients with INR values above the recommended range for anticoagulation form a separate group in the upper left corner. The positions of the variables in the loadings plot suggest that most patients with high INR values are taking doses of warfarin and have similar concentrations of warfarin in the oral fluid to patients with INR in the recommended therapeutic range. The main exception is patient P9, who falls in the upper right corner for taking high doses of warfarin and shows very high concentrations of the drug in the oral fluid. The loadings plot also suggests the existence of a positive correlation between dose and warfarin concentration in the oral fluid as well as a negative correlation between INR and pH values.

**Figure 3:**
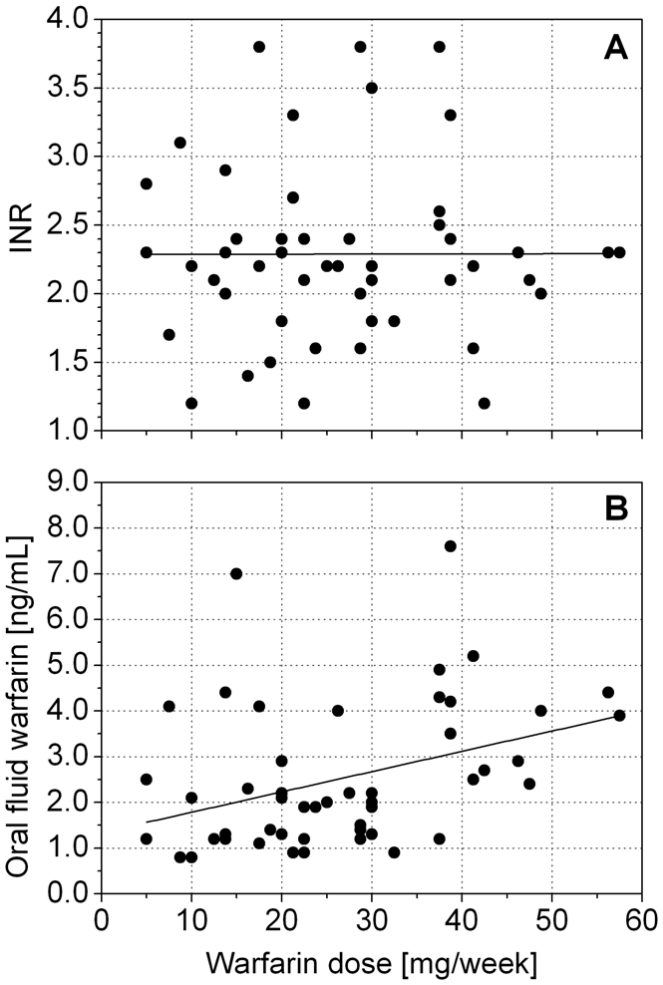
Effect of the warfarin weekly dose on A) INR and B) warfarin concentration in oral fluid.

Figure 3 reports INR and warfarin concentration values in oral fluid versus weekly dose. No correlation was found between INR values and dose (r = −0.03, p = 0.85, Fig. 3A), whereas there was a clear positive correlation between warfarin concentration in the oral fluid and dose (r = 0.39, p = 0.006, Fig. 3B).

The first result reflects the wide variability of patient responses to the drug observed in over fifty years of clinical use. This variability has been ascribed to factors such as gastrointestinal absorption, age, renal and hepatic function, lifestyle (in particular diet, alcohol consumption, smoking etc.) [6], [26]. The concomitant assumption of other drugs can change the fraction of warfarin bound to plasma proteins and influence its pharmacokinetics and pharmacodynamics. Genetic variability in the cytochrome P450 (for specific CYP isoforms) has also been identified as a further key factor [27]–[29].

The correlation between warfarin concentration in oral fluid and dosage is also not surprising. Warfarin concentration in oral fluid is expected to mirror (if the pH level in the oral fluid is not much lower than plasma pH) the concentration of free warfarin in plasma, and to be highly correlated to the total warfarin concentration in plasma. The ratio between the concentrations of bound and unbound warfarin in plasma, in fact, is reported to be generally subject to only minor variations (between 0.01 and 0.03), with the exception of the concomitant assumption of other drugs capable of displacing bound warfarin from albumin [30]. Obviously, the degree of correlation between warfarin concentration in oral fluid and dosage cannot be higher than the correlation existing between warfarin concentration in plasma and dosage. A correlation coefficient of r = 0.,55 was found by Lombardi et al. [31] between total warfarin concentration in plasma and weekly dosage, whereas Huang et al. [32] found a correlation coefficient of 0.378 between the concentration of free warfarin in plasma and weekly dosage. Our value is compatible with these results.

**Figure 4:**
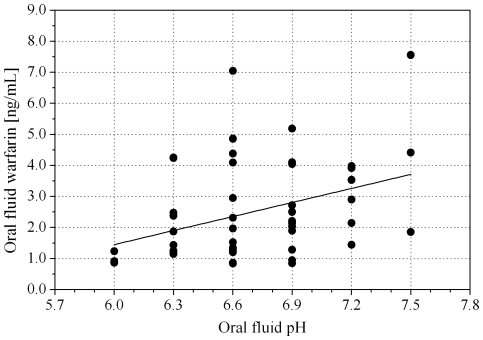
Correlation between the concentration of warfarin and pH in the oral fluid.

A correlation was found between the concentration of warfarin in the oral fluid and the pH value (r = 0.37, p = 0.009) (Fig. 4). We believe that this correlation may be due to a hindered warfarin transfer from plasma when pH values of the oral fluid approach the pK_a_ of warfarin (5.19). In fact the lowest warfarin concentration levels in oral fluid were observed in the most acidic samples. Two patients, with pH values of oral fluid lower than 6, had to be excluded from the study because the warfarin concentration in the oral fluid was below the quantification limit.

**Figure 5:**
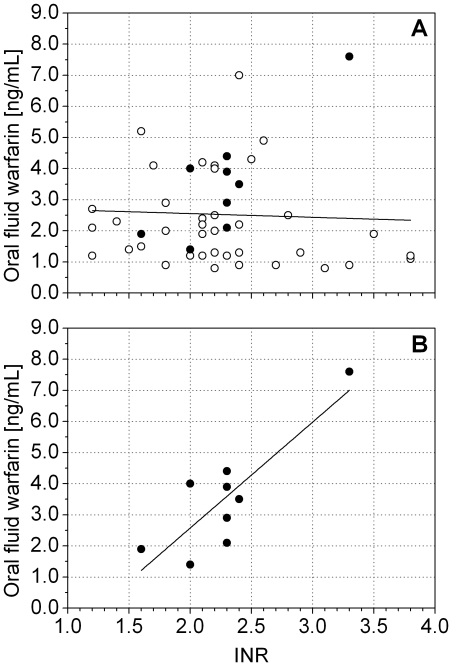
Warfarin concentration in oral fluid versus INR. Samples with a pH level higher than 7.2 are indicated by full dots: A) all the samples, B) detail of samples with a pH level higher than 7.2.

Figure 5A reports the warfarin concentration in oral fluid versus INR. No correlation was found between these two parameters (r = −0.11, p = 0.43). However, if only the samples with pH values equal or higher than 7.2 were considered, a correlation between these two parameters was observed (r = 0.84, p = 0.004, Fig. 5B). Although the limited number of patients does not enable a firm conclusion to be drawn on this point, the result is compatible with the hypothesis that there is a good correlation between the free warfarin concentration in plasma and INR, which is only mirrored in the oral fluid when its pH value enables the drug to be transferred from the blood.

**Figure 6:**
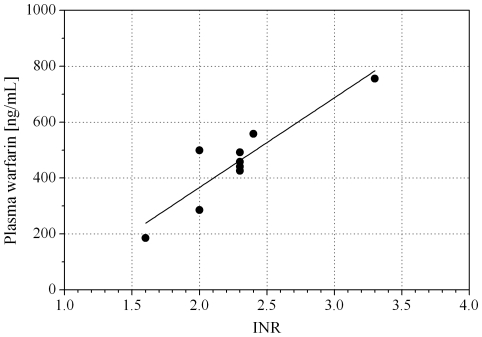
Correlation between calculated concentration of warfarin in plasma and International Normalized Ratio for patients with a pH≥7.2.

Huang et al. [32] found a modest but significant correlation between free warfarin in plasma and INR (r = 0.207, p = 0.03), as did Lombardi et al. [31] who found a similar correlation between total warfarin in plasma and INR (r = 0.25, p = 0.079). In comparison with these studies, our set of patients showed a lower correlation between warfarin concentration in oral fluid and INR when the pH of oral fluid was lower than plasma pH, however they showed a higher correlation when these pH values were in the same range. This suggests that when pH conditions lead to an optimal diffusion from blood, the warfarin assayed in the oral fluid may reflect the pharmacodynamics more precisely, thus maybe overcoming the effect of an unknown confounding factor connected to the complex chemical nature of blood. To confirm this second hypothesis, we used measured pH values to calculate the theoretical warfarin concentration values in plasma by the Rasmussen equation (Fig. 6) [21]. The use of the pH information resulted in an increased correlation (r = 0.91, p = 0.0006).

In summary, we have presented a method for analysing warfarin in oral fluid which highlights the presence of the drug in both oral fluid and sweat at concentration levels of a few ng/mL. The value of pH in oral fluid is likely to play an important role in determining the transfer of the drug from blood. The correlation between the concentration of warfarin in the oral fluid and INR was similar to the correlation between warfarin concentrations in plasma and INR reported in previous studies. However, we found a much higher correlation between oral fluid warfarin and INR in patients with an oral fluid pH above 7.2, which might be of a potential clinical interest. When the Rasmussen equation was used to estimate the plasma warfarin concentration in this group of patients, the resulting correlation with INR values increased significantly.

The variability of the oral fluid pH within the same individual over time and among different individuals, its role in determining the warfarin transfer rates and the possibility of using its value to predict warfarin concentrations in blood need to be clarified prior to any clinical application. The determination of warfarin concentration in oral fluid could become a noninvasive test enabling the efficacy and safety of the therapy to be monitored in the time span between two conventional INR tests. In fact, due to the 72-hour delay in the biological action of warfarin, an increase in concentration in oral fluid could be an early signal of risk of hemorrhagic event. Such tests could be particularly useful in patients with high levels of anticoagulation or undergoing a combined anticoagulation/antiaggregation treatment.

## Supporting Information

Figure S1 **Chromatograms of representative sweat samples.** A) a blank sweat sample from a volunteer not taking the drug; B) a patient sweat sample with an estimated warfarin concentration of about 4 ng/mL (t_r_ = 10.2 min).
(TIF)

Figure S2 **Distribution of pH values in patient oral fluid samples.**
(TIF)
