# Supplementary figures and images for: Measurement of Warfarin in the Oral Fluid of Patients Undergoing Anticoagulant Oral Therapy
Source: PLoS One. 2011 Dec 2;6(12):e28182. doi: 10.1371/journal.pone.0028182 (PMC3229510; doi:10.1371/journal.pone.0028182)

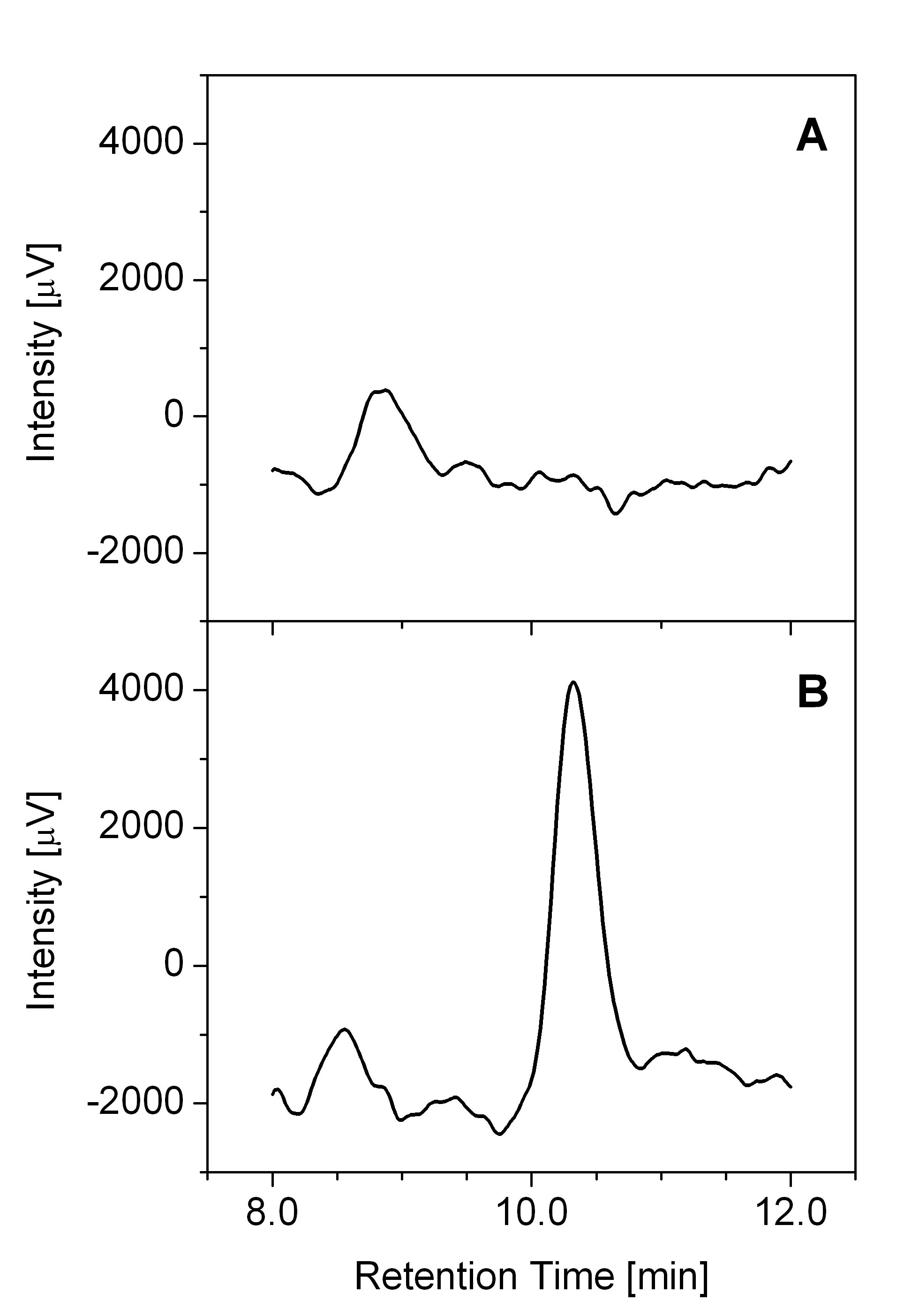

Supplement: Figure S1 — Chromatograms of representative sweat samples. A) a blank sweat sample from a volunteer not taking the drug; B) a patient sweat sample with an estimated warfarin concentration of about 4 ng/mL (tr = 10.2 min). (TIF) [file pone.0028182.s001.tif]

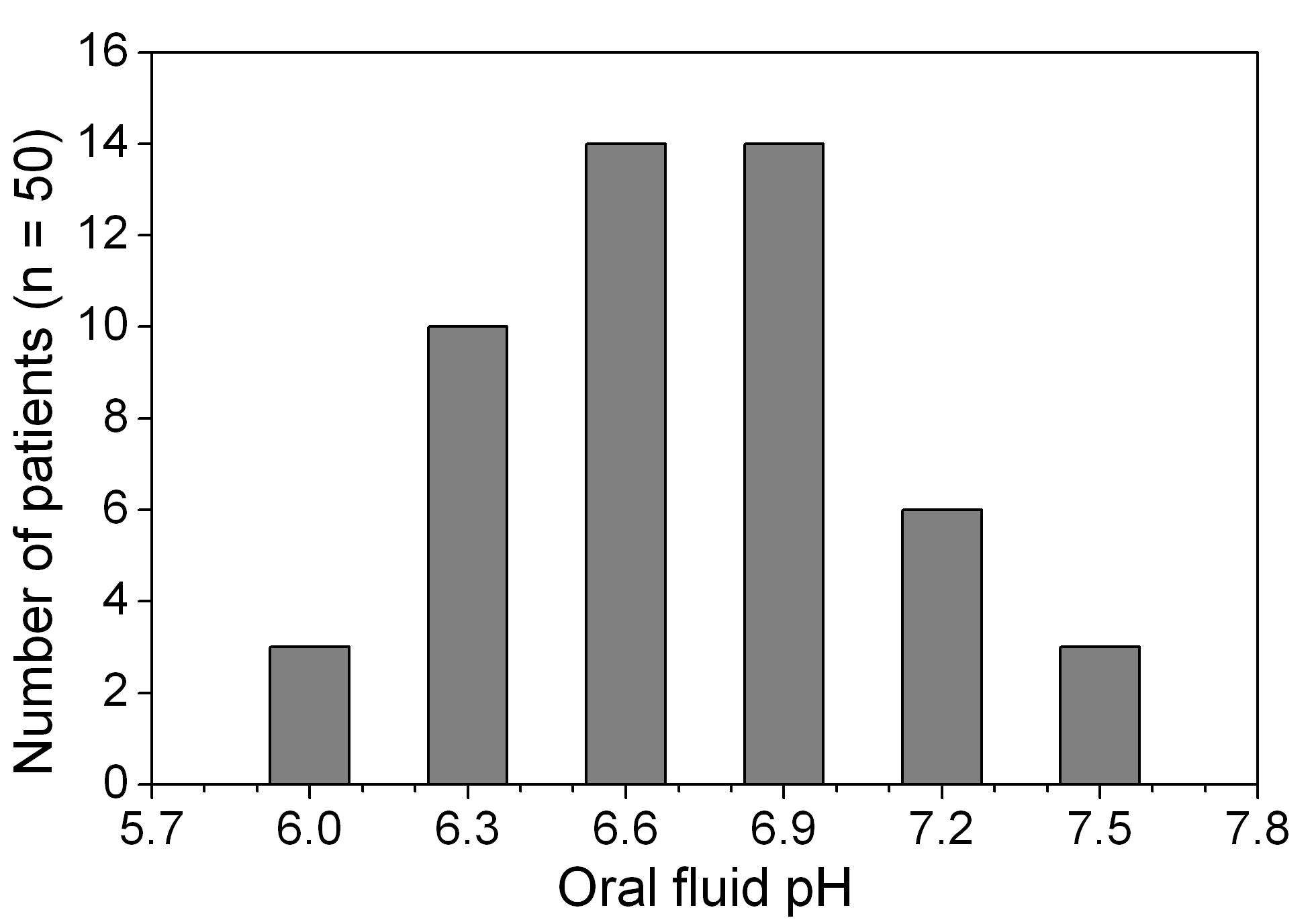

Supplement: Figure S2 — Distribution of pH values in patient oral fluid samples. (TIF) [file pone.0028182.s002.tif]
